# Liberal or conservative oxygen therapy for ventilated patients in the ICU: a meta-analysis of randomized controlled trials

**DOI:** 10.1186/s13019-021-01634-4

**Published:** 2021-09-15

**Authors:** Lu Liu, Yali Tian

**Affiliations:** 1 Department of Anesthesiology, West China Hospital/West China School of Nursing, Sichuan University, Chengdu, 610041 China; 2 West China School of Nursing, Sichuan University/West China Hospital, Sichuan University, Chengdu, 610041 China

**Keywords:** Oxygen saturation, ICU, Meta-analysis

## Abstract

**Background:**

The acknowledgment that conservative oxygen therapy (COT) was related to better prognosis in the intensive care unit (ICU) was challenged recently. We conducted an updated meta-analysis aimed to determine whether liberal oxygen therapy (LOT) or COT is associated with better improve clinical outcomes.

**Methods:**

We systematically searched the electronic databases (PubMed, Web of Science and Embase) up to May 2021 for randomized controlled trials (RCTs). The primary outcome was the mortality of the final follow-up time and secondary outcomes were ICU mortality, the ICU length of stay and the number of ventilator-free days.

**Results:**

A total of 7 RCTs were included, with 2166 patients admitted to the ICU. There was no significant difference in the primary outcome between the LOT and COT. Additionally, LOT could not significantly increase ICU mortality and the ICU length of stay compared with COT.

**Conclusions:**

The present study showed that COT was not significantly superior to LOT in clinical outcomes. Therefore, additional high-quality studies with novel designs are required to further elucidate this controversy.

## Background

In critically ill patients, the provision of supplemental oxygen is universal to patients who require invasive mechanical ventilation in the intensive care unit (ICU) [1]. The use of supplemental oxygen is aimed to prevent or reverse hypoxemia. The liberal oxygen therapy (LOT) may provide a baseline of safety against hypoxia [2, 3]. However, excess oxygen delivery could expose patients to hyperoxia that leads to potential iatrogenic harm, such as pulmonary injury, interstitial fibrosis, central nervous system toxicity, etc. [2, 4–6]. Conservative oxygen therapy (COT) could minimize the chance of exposure to high levels of oxygen and reduce the occurrence of hyperoxia [7]. In a previous meta-analysis of randomized trials about acutely ill adults, COT has been proved to be associated with lower in-hospital mortality compared with LOT [8].

Notably, several recent randomized controlled trials (RCTs) did not support the superiority of COT over LOT in ICU patients. Mackle et al. and Barrot et al. suggested that implementation of conservative-oxygenation strategy did not significantly affect the number of ventilator-free days and decrease the mortality rate when compared with LOT [7, 9]. Another RCT demonstrated that COT did not significantly improve the prognosis when compared to LOT in ICU patients with sepsis. The point estimate of treatment effect even preferred the LOT approach [10].

Therefore, giving the fact that several RCTs comparing LOT versus COT for ICU patients suggested conflicting results, we conducted an updated meta-analysis of RCTs involving ICU patients to compare LOT versus COT and synthesized the prognosis results.

## Methods

### Search strategies

The literature search was performed in Pubmed, Embase, and MEDLINE by combining the following keywords: (“conservative oxygen therapy” or “COT”), (“conventional oxygen therapy” or “liberal oxygen therapy” or “LOT” or “usual oxygen therapy”), (“ICU” or “intensive critical care” or “critical care”), and (“RCT” or “Randomized Controlled Trial” or “Controlled clinical trial” or “Random*”). In addition, the references of related articles were searched manually for studies if missed in the database searches.

### Inclusion and exclusion criteria

The included studies in the meta-analysis met the following criteria: (1) randomized controlled trials; (2) subjects in studies were patients admitted in ICU who were expected to remain mechanically ventilated; (3) the patients were assigned to receive either COT or LOT; (4) the outcomes included deaths in final follow-up time, ICU mortality, length in ICU days, and mechanical ventilation-free days. The exclusion criteria were as follows: (1) meta-analyses, reviews, case reports, and protocols; (2) non-English articles. Two reviewers (LL, YT) independently screened the titles and abstracts following the criteria and review the full text of eligible studies to determine the final inclusion. Any difference was resolved with a third reviewer.

### Data extraction

Data from the included studies were extracted by two reviewers (LL, YT) independently. A third reviewer was ready to adjudicate any unsolved disagreements. The following variables were extracted: the first author’s name, the country of study, the publication year, the study design, population, follow-up duration, in-hospital details, such as type of admission to ICU, median PaO_2_ and SpO_2_, and severity scores: the acute physiology and chronic health evaluation score-II (APACHE II); the simplified acute physiology score-II and III (SAPS II and SAPS III); sequential organ failure assessment (SOFA); intervention features, death in final follow-up time as the primary outcome, and secondary outcomes including ICU mortality, length in ICU days, and mechanical ventilation-free days.

### Quality assessment

The risk of bias of each study was assessed by two independent reviewers using methods from The Cochrane Collaboration [11], which require response one of the “low risk”, “high risk”, or “unclear” to the following criteria as indicators of the quality of trials: (1) selection bias, including random sequence generation and allocation concealment; (2) performance bias or blinding of participants and personnel; (3) detection bias or blinding of outcome assessment; (4) attrition bias or incomplete outcome data; (5) detection attrition bias or incomplete outcome data; (6) reporting bias or selective reporting, and (7) other bias. Disagreements were adjudicated by the third reviewer.

### Statistical analysis and assessment of heterogeneity

We performed all statistical analyses in R (version 4.0.3). Heterogeneity between studies was examined by using the I^2^ statistic. The magnitude of heterogeneity was considered as low, moderate, and high by I^2^ values of 25%, 50%, and 75%. When I^2^ > 50%, a random-effects model was applied in our meta-analyses. Otherwise, we used the fix-effects model. For dichotomized outcome data, such as mortality, we calculated the risk ratios (RRs) with the 95% confidence intervals (CIs), while we calculated mean differences (MDs) with the 95% CIs for continuous outcome data. Subgroup analyses were performed for the primary outcome according to the follow-up time and the publication year. In addition, we carried out the contour-enhanced funnel plots to examine the publication bias.

## Results

### Literature search

**Fig. 1:**
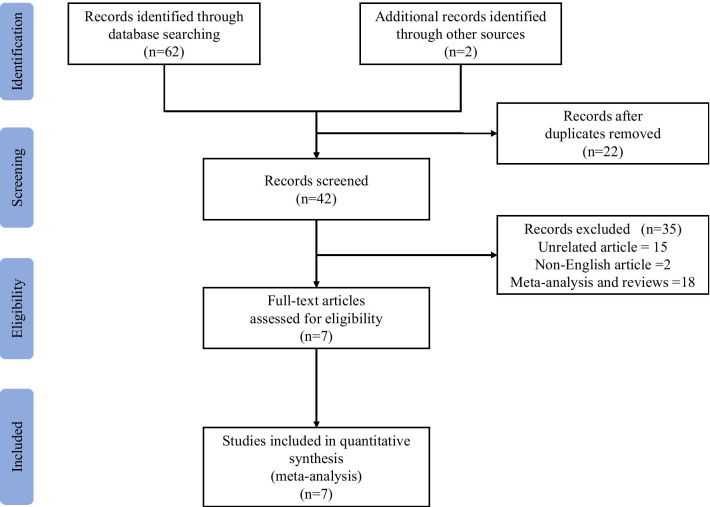
The flowchart for the selection of studies

Sixty-four studied were identified by the literature search. After screening all the studies, 57 articles were excluded, including 22 duplications, 15 unrelated articles, 18 meta-analyses and reviews, and 2 non-English articles. Seven studies in total were included in this meta-analysis [7, 9, 10, 12–15] (Fig. 1).

### Study characteristics

The included studies were published between 2014 and 2020 from 5 countries. There were 2166 patients from 7 studies included in our meta-analysis, 1077 of which received COT and 1089 patients received LOT. One study focused on children and other studies focused on adult patients. The details of each study were shown in Table 1.

**Table 1:** The detailed characteristics of the patients at baseline between the liberal and conservative oxygen therapy

Study	Country	Design	Intervention	Patient						
				Mean age	SD	Male	Sample size	Follow-up duration	Spo_2_	Pao_2_
Young [10]	New Zealand	Multicenter	LOT	58.3 ± 15	15	75	130	90 days, 180 days	96.2% ± 3%	97 ± 61.9
			COT	57.2 ± 14.3	14	59	121		95.9% ± 3.7%	99 ± 50.5
Mackle [7]	Australian and New Zealand	Multicenter	LOT	58.1 ± 16.2	16	306	484	90 days, 180 days	97.1% ± 3.1%	110 ± 69.6
			COT	57.5 ± 16.1	16	302	481		96.7% ± 3.7%	112 ± 63
Barrot [9]	French	Multicenter	LOT	63 ± 15.5	16	65	99	28 days, 90 days	–	90.3 ± 38.8
			COT	63.5 ± 14.5	15	64	102		–	92.3 ± 44.8
Peters [12]	UK	Multicenter	LOT	1.9 (0.4,5)	3.4	21	54	–	99% ± 2.22%	–
			COT	0.8 (0.1,2)	1.4	29	53	–	99% ± 1.48%	–
Girardis [14]	Italy	Single-center	LOT	65 ± 17.8	18	125	218	24 days	–	–
			COT	63 ± 17	17	121	216	22 days	–	–
Panwar [13]	Australia	Multicenter	LOT	NA	NA	NA	52	90 days	93% ± 2%	72 ± 10
			COT	NA	NA	NA	51		97 ± 1%	95 ± 15
Suzuki [15]	Australia	Single-center	LOT	56 ± 16	16	32	54	28 days	95.5% ± 2.44	83 ± 17
			COT	59 ± 17	17	38	51		98.4% ± 1.33%	107 ± 27.4

Study	Patient						Type of admission to ICU			
	Pao_2_/Fio_2_ ratio	PEEP	APACHE II	SAPS III	SAPS II	SOFA	Emergency	Hospital ward	Surgery	Others (from another ICU or hospital)
Young [10]	213 ± 118	–	22.7 ± 7.5	–	–	–	43	44	32	11
	200 ± 102	–	22.8 ± 8.2	–	–	–	50	34	24	13
Mackle [7]	259 ± 146	6 ± 3.7	23.6 ± 9.3	–	–	–	181	107	149	41
	245 ± 138	6 ± 3.7	23.3 ± 9.4	–	–	–	212	82	146	41
Barrot [9]	116.8 ± 47.7	6.2 ± 2.7	–	66.9 ± 13.7	–	9.3 ± 3.68	–	–	–	–
	120.1 ± 53.6	6.4 ± 3.5	–	67.9 ± 14.4	–	8.9 ± 3.6	–	–	–	–
Peters [12]	–	–	–	–	–	–	–	–	–	–
	–	–	–	–	–	-	–	–	–	–
Girardis [14]	–	–	–	–	37 (26–49)	–	–	86	132	–
	–	–	–	–	39 (28–55)	–	–	77	139	–
Panwar [13]	–	–	–	–	–	–	–	–	–	–
	–	–	–	–	–	–	–	-	–	–
Suzuki [15]	177 ± 91.1	5.2 ± 1.78	62 ± 31.9	–	–	–	–	–	–	–
	158 ± 86.7	6.1 ± 1.85	68 ± 38.5	-	–	–	–	–	–	–

*LOT* liberal oxygen therapy, *COT* conservative oxygen therapy, *SD* standard deviation, *SpO*_*2*_ arterial saturation of peripheral oxygen, *PaO*_*2*_ partial pressure of oxygen, *PaO*_*2*_*/FiO*_*2*_ partial pressure of oxygen/ Fraction of inspire oxygen, *PEEP* positive end-expiratory pressure, *APACHE II* The acute physiology and chronic health evaluation score-II, *SAPA III* The simplified acute physiology score-III, *SAPA II* The simplified acute physiology score-II, *SOFA* sequential organ failure assessment

### Mortality in final follow-up and subgroup analysis

**Fig. 2:**
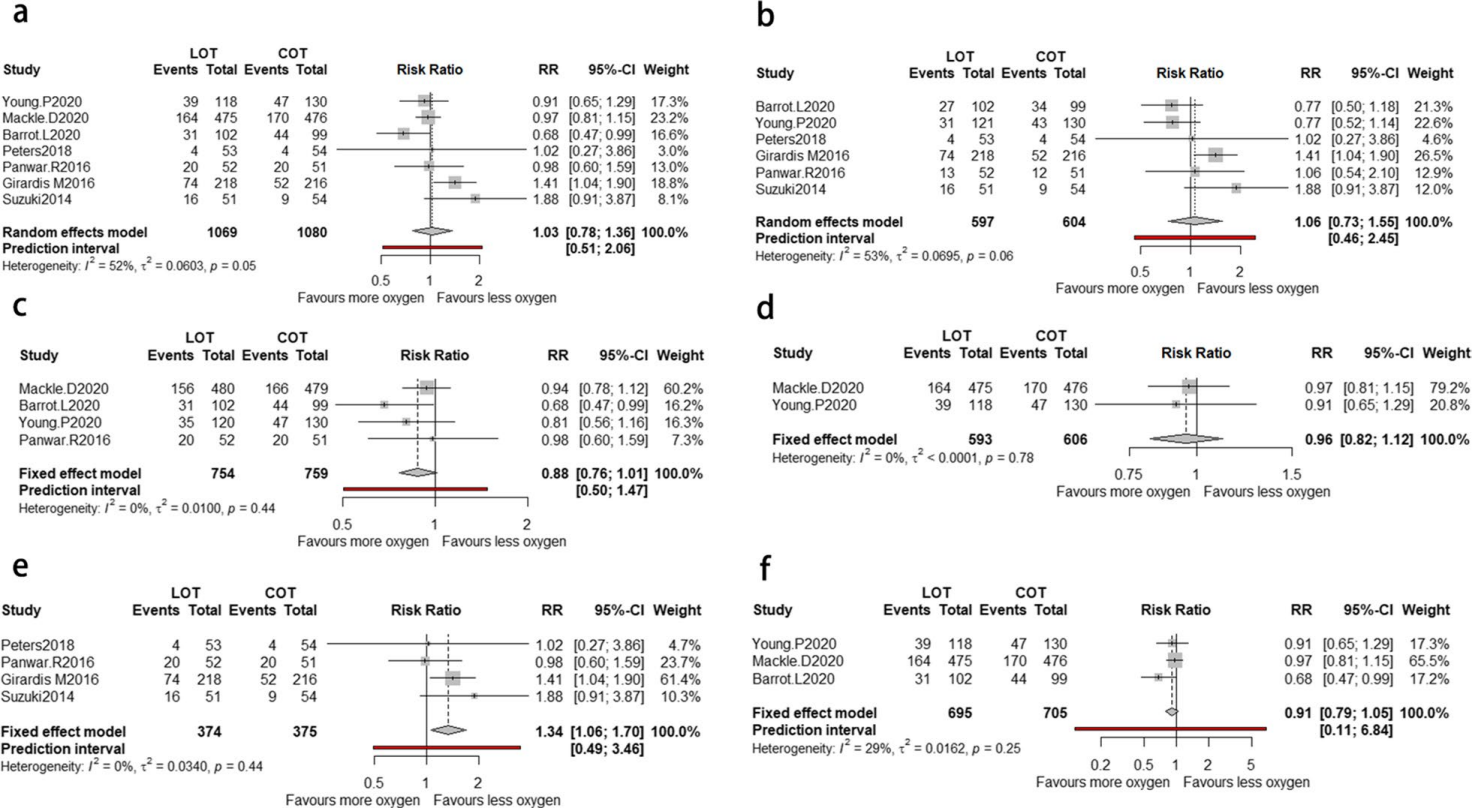
The forest plot of mortality between LOT and COT **a** The longest follow-up time. **b** Within 90-days. **c** In 90-days. **d** In 180-days. **e** According to the studies published before 2020. **f** According to the studies published after 2020 between LOT and COT. *LOT* liberal oxygen therapy, *COT* conservative oxygen therapy, *RR* relative risk

Mortality in the longest follow-up did not have significant difference between LOT and COT groups, with low heterogeneity (RR = 1.03; 95% CI [0.78; 1.36], I2 = 35%) (Fig. 2a). In the subgroup analysis, the results of mortality within 90-days (RR = 1.06; 95% CI [0.73; 1.55], I2 = 53%, Fig. 2b) and 180-days mortality (RR = 0.96; 95% CI [0.82; 1.12], I2 = 0%) were not significant (Fig. 2c). However, the result of 90-days mortality (RR = 0.88; 95% CI [0.76; 1.01], I2 = 0%, Fig. 2d) strongly favored the LOT, though not significant. When performing subgroup analysis according to the publication date, we found that COT significantly associated with reduced mortality based on the studies published before 2020 (RR = 1.34; 95% CI [1.06; 1.70], I2 = 0%, Fig. 2e), while the result was insignificant when it comes to the studies published after 2020 (RR = 0.91; 95% CI [0.79; 1.05], I2 = 29%) (Fig. 2f).

### ICU mortality

**Fig. 3:**
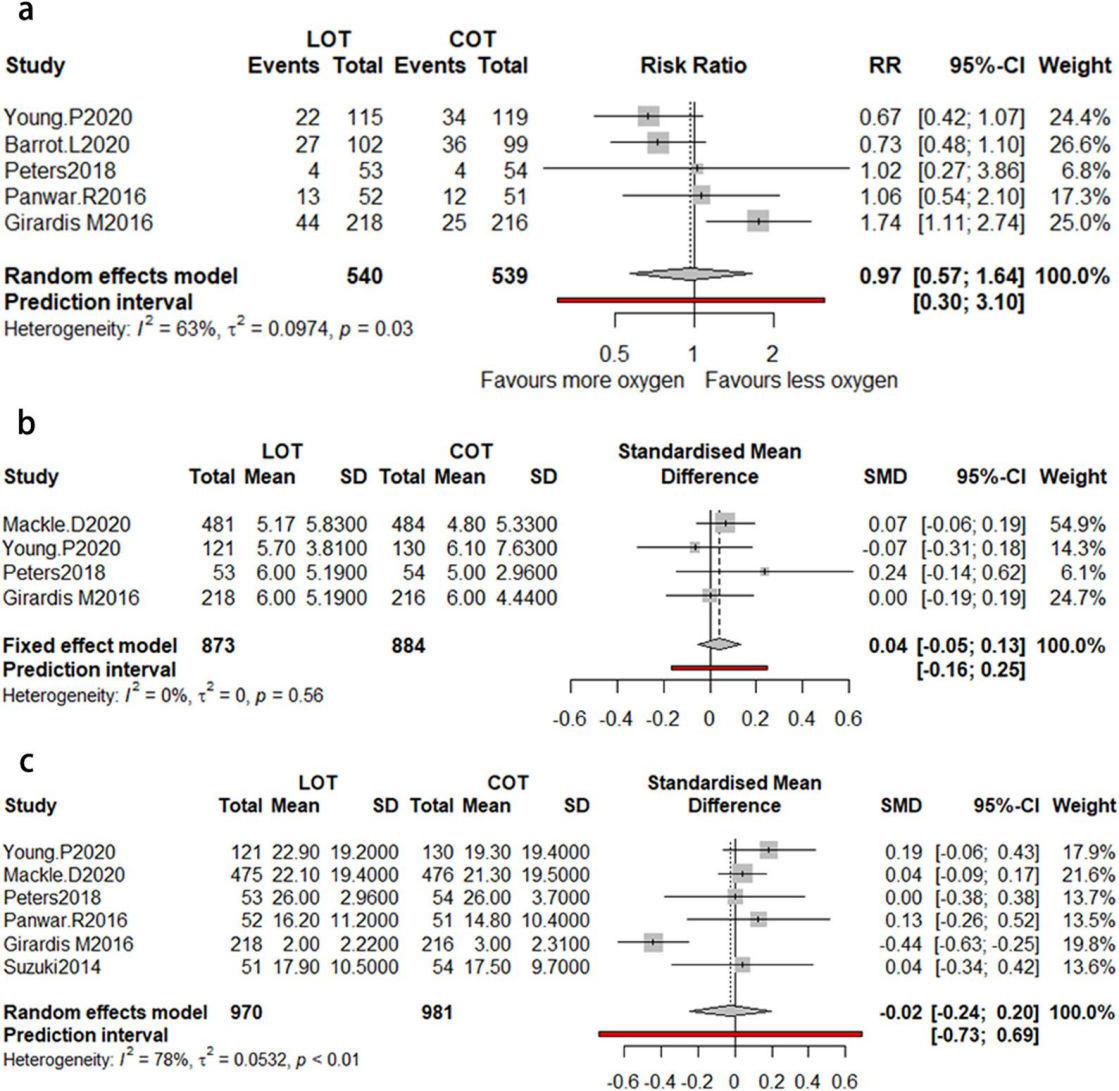
**a** The forest plot of mortality in ICU between LOT and COT. **b** The forest plot of the length of ICU days between LOT and COT. **c** The forest plot of the mechanical ventilation-free days between LOT and COT. *LOT* liberal oxygen therapy, *COT* conservative oxygen therapy, *RR* relative risk

A total of five studies provided available data with 1079 patients for ICU mortality (Fig. 3a). The meta-analysis showed that no significant correlation was found between ICU mortality and the two types of oxygen therapy, with moderate heterogeneity (RR = 0.97; 95% CI [0.57; 1.64], I2 = 63%).

### ICU length of stay

A total of four studies showed relevant data of 1757 patients for ICU median days (Fig. 3b). We did not find significant difference between LOT and COT group, with low heterogeneity (SMD = 0.04; 95% CI [− 0.05; 0.13], I2 = 0%).

### MV-free days

Six studies in total provided relevant data of 1951 patients for MV-free days (Fig. 3c). The pooled analysis showed that LOT was significantly related to reduced MV-free days compared with COT (SMD = − 0.02; 95% CI [− 0.24; − 0.20], I2 = 78%).

### Publication bias

**Fig. 4:**
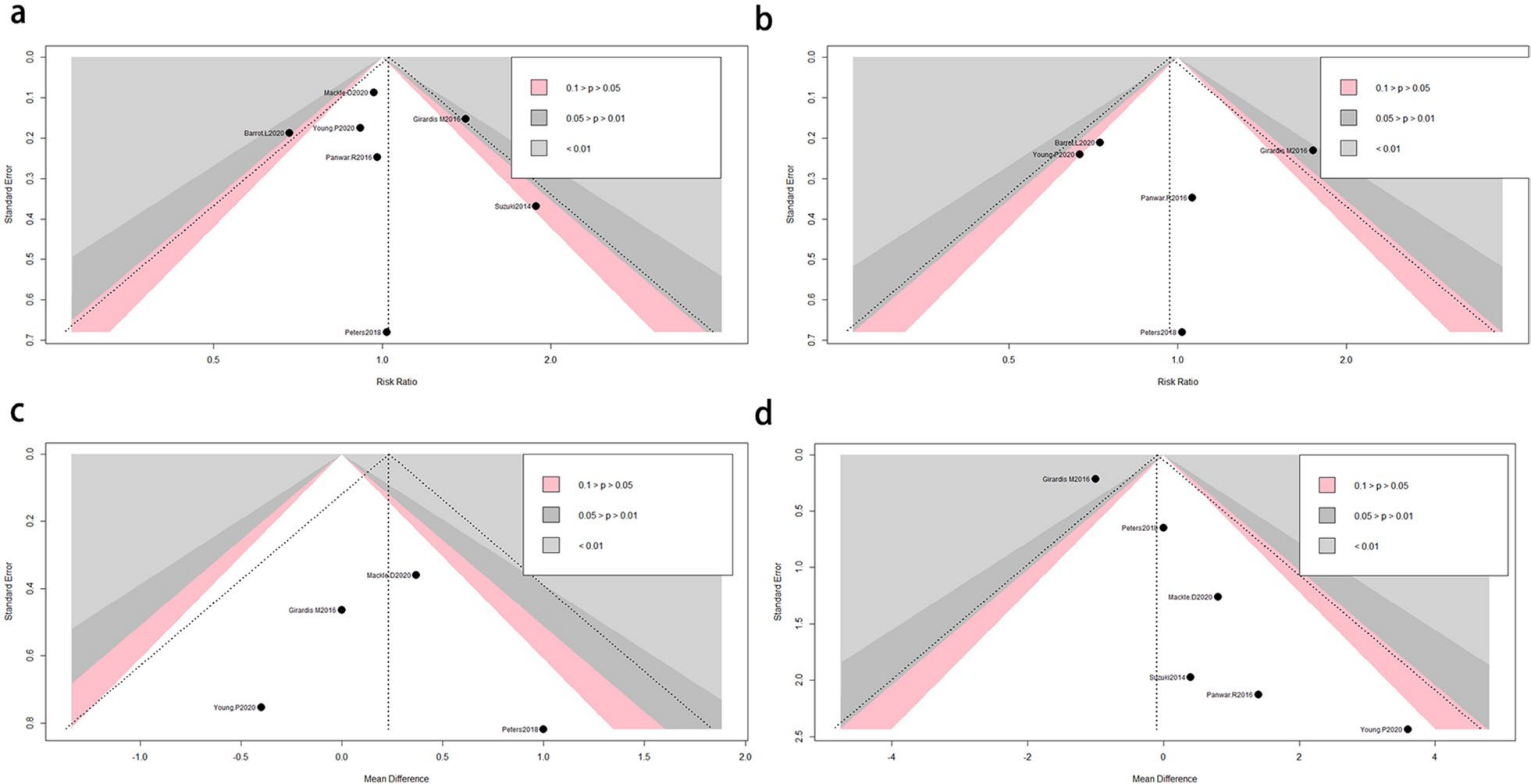
**a** The funnel plot for the death in final follow-up time. **b** The funnel plot for the ICU mortality. **c** The funnel plot for the length in ICU days. **d** The funnel plot for the mechanical ventilation-free days. *LOT* liberal oxygen therapy, *COT* conservative oxygen therapy, *ICU* intensive care unit, *RR* relative risk

Funnel plots were performed to examine the publication bias of included studies (Fig. 4). We found no significant publication bias for the longest follow-up mortality, ICU mortality, ICU length of days, and MV-free days.

### The methodological quality of studies

**Fig. 5:**
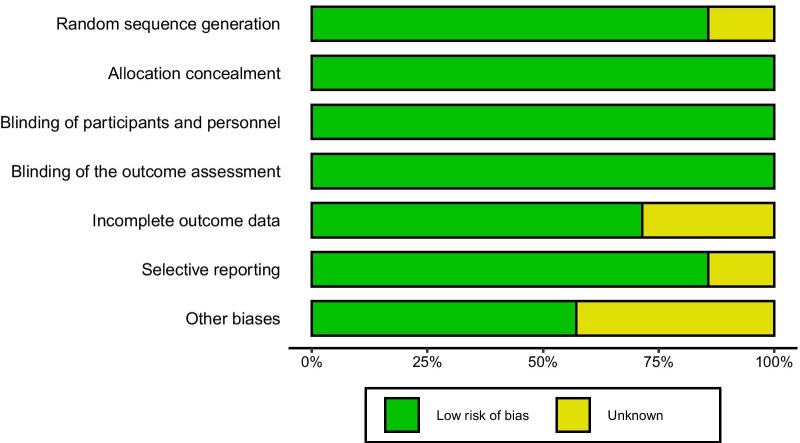
The summary of the risk of bias of the included randomized controlled trials. The green region represents a low risk of bias. The yellow region represents unclear bias

The overall risk of bias of included studies is low (Fig. 5). The attrition bias of the two studies was “unclear”, while others were assessed as “low risk”. All studies had a “low risk” of reporting bias, except one with “unclear”. In the assessment of other biases, three studies had a quality indicator that was “unclear”, while others had “low risk”.

## Discussion

The key finding of this meta-analysis was that the superiority of COT over LOT was challenged for patients admitted to ICU with the publishment of high-quality RCTs in 2020. In subgroup analysis, we also did not find significant differences in less than 90-day, 90-day, and 180-day mortality rates between the two therapies. We even observed the trend supporting a lower 90-day mortality rate in the LOT group.

Whereas in previous studies, meta-analyses of observational studies [16, 17] and RCTs [8] from various critical care settings and groups of patients have shown that LOT was associated with increased mortality risk in critically ill adults. Besides the reason mentioned in the previous section (LOT increases the probability of harm from hyperoxia), it might also be that excessive supplemental oxygen could lead to falsely reassuring SpO_2_ values in clinical practice [18, 19]. This might lessen clinician vigilance and delay the optimal treatment for patients. On the other hand, the fundamental diseases of patients also have major influences on the results. As illustrated previously, arterial hyperoxia was significantly related to the mortality in patients resuscitated from cardiac arrest but not in mechanically ventilated patients [16]. The reason could be hyperoxia-induced vasoconstriction and cardiac output reduction [16, 17].

To further illustrate the change of effect in LOT and COT to ICU patients, we conducted subgroup analysis according to follow-up time and publication year. Before 2020, the 4 articles we involved demonstrated that LOT could significantly result in more mortality than COT. These two papers [14, 15] contributed the most to the effect size. But the findings of 3 articles published after 2020 are at variance with the previous results. Young et al. found that the 90-day mortality of sepsis patients in ICU who received LOT was 7 percentage points lower than that of the COT group, although not significant [10]. For patients with acute respiratory distress syndrome (ARDS), Barrot et al. demonstrated that the 90-day mortality rate in LOT group was 14 percentage points lower than that in the COT group [9].

Several possible factors might explain the results. First, the patients’ characteristics should be an important concern. For patients with sepsis, oxygen delivery to the tissues could be impaired and excess oxygen delivery might help to reverse this situation and avoid cellular and organ dysfunction [10]. While for patients with hypoxic-ischemic encephalopathy, it is biologically plausible that COT reduces the incidence of secondary brain damage after resuscitation from cardiac arrest [7]. Moreover, based on the APACHE II score, the disease state of patients included in the Suzuki et al. [15] was severer than that in the Young et al. [10] and Mackle et al. [7]. Second, from the study design perspective, the follow-up duration of these two studies [14, 15] are relatively shorter than the studies published after 2020 and they are single-center studies. Third, targeting lower oxygenation might decrease oxygen content and transport and liberal use of oxygen may provide a baseline of safety against hypoxia in the long run.

This study also had some limitations. Firstly, the time of intervention, the duration of mechanical ventilation, and the definitions and implementation of LOT and COT are hard to be unified and impractical to be consistent in clinical work. It was also reported that clinicians might prone to switch from controlled ventilation to a mode allowing unassisted ventilation in the presence of a lower fraction of inspired oxygen (Fio_2_). Secondly, the methods of information gathering between publications could be diverse. Some papers are short of clinical parameters such as lactate and central venous oxygen saturation, which could be major influences during the treatment.

## Conclusions

In conclusion, recent clinical trials targeting ICU patients showed that COT, as compared with LOT, did not significantly decrease the long-term and short-term mortality.

## Data Availability

The datasets used and/or analyzed during the current study are available from the corresponding author on reasonable request.

## Abbreviations

LOT: Liberal oxygen therapy
COT: Conservative oxygen therapy
RCTs: Randomized controlled trials
ICU: Intensive care unit
MV: Mechanical ventilation
CIs: Confidence intervals
MDs: Mean differences
RRs: Risk ratios
ARDS: Respiratory distress syndrome
APACHE II: The acute physiology and chronic health evaluation score-II
SAPA III: The simplified acute physiology score-III
SAPA II: The simplified acute physiology score-II
SOFA: Sequential organ failure assessment
